# Solid- and Vapor-Phase Antibacterial Activities and Mechanisms of Essential Oils Against Fish Spoilage Bacteria

**DOI:** 10.3390/antibiotics13121137

**Published:** 2024-11-26

**Authors:** Hsuan-Ju Lin, Pang-Hung Hsu, Tze-Chia Lin, Wen-Jung Lu, Hong-Ting Victor Lin

**Affiliations:** 1 Department of Food Science, National Taiwan Ocean University, No. 2, Pei-Ning Road, Keelung 202, Taiwan; angel810801@gmail.com (H.-J.L.); miss350100@gmail.com (W.-J.L.); 2 Department of Bioscience and Biotechnology, National Taiwan Ocean University, No. 2, Pei-Ning Road, Keelung 202, Taiwan; phsu@ntou.edu.tw; 3 Center of Excellence for the Oceans, National Taiwan Ocean University, No. 2, Pei-Ning Road, Keelung 202, Taiwan; 4 Institute of Biochemistry and Molecular Biology, National Yang Ming University, No. 155, Sec. 2, Linong Street, Taipei 112, Taiwan; 5 K. F. Lings Co., Ltd., No. 294, Furong Street, Taoyuan 334, Taiwan; tzechia.lin@gmail.com; 6 School of Life Sciences, Queen’s Medical Centre, University of Nottingham, Nottingham NG7 2UH, UK

**Keywords:** essential oil, seafood spoilage bacterium, antibacterial activity, vapor-phase antibacterial activities, proteomic analysis

## Abstract

Essential oils (EOs), regarded as secondary metabolites from plants, possess effective antibacterial properties. This study investigates the antibacterial efficacy of seven citrus EOs against six spoilage bacteria: *Vibrio parahaemolyticus*, *V. harveyi*, *Photobacterium damselae*, *Shewanella putrefaciens*, *Carnobacterium divergens*, and *Lactobacillus pentosus*. The antibacterial activity of these EOs was evaluated using solid- and vapor-phase applications. All tested EOs demonstrated effective antibacterial activity at a concentration of 294 μL/L against Gram-negative bacteria. Notably, lemon and orange EOs exhibited dose-dependent inhibition in both solid- and vapor-phase applications, with minimum effective concentrations ranging from 29.4 to 58.8 μL/L. Following treatment with lemon and orange EOs for 6 h at 1/4 minimum inhibitory concentration, leakage of intracellular DNA and proteins was observed, indicating damage to the cell membrane/wall. Proteomic analysis revealed distinct mechanisms: lemon EO impaired bacterial antioxidant defenses, while orange EO disrupted cell division, leading to reduced bacterial viability. These findings provide valuable insights into the potential of different EO application forms in controlling spoilage bacteria.

## 1. Introduction

Essential oils (EOs) are volatile, aromatic compounds found in various parts of plants, including flowers, leaves, seeds, bark, and roots. They are used widely in industries like cosmetics, medicine, and food preservation due to their pleasant scents and practical benefits [1]. Recently, EOs have gained more attention for their therapeutic potential and diverse biological activities [2,3].

EOs show antibacterial activity against both Gram-positive and Gram-negative bacteria, making them valuable in food preservation. This broad-spectrum antibacterial effect is due to their rich chemical composition, which includes compounds like terpenes, phenolics, aldehydes, and alcohols. These compounds disrupt bacterial cell membranes, increasing permeability and interfering with essential functions like energy production and nutrient transport [4]. Specific components, such as thymol, eugenol, and carvacrol, have been shown to inhibit bacterial enzymes, DNA replication, and protein synthesis, ultimately leading to bacterial death [5,6].

Aquatic products like fish and shellfish spoil quickly due to their high moisture content and nutrient-rich composition, creating an ideal bacterial growth environment. Some key bacteria involved in seafood spoilage include *Photobacterium damselae*, *Shewanella putrefaciens*, *Carnobacterium divergens*, *Lactobacillus pentosus*, *Vibrio parahaemolyticus*, and *V. harveyi*. These bacteria break down proteins and fats, causing odors, discoloration, and texture changes that reduce product quality and shelf life. For example, *S. putrefaciens* is involved in sulfurous odors and slime formation in spoiled fish, while *V. parahaemolyticus* and *V. harveyi* contribute to rapid spoilage and foodborne illnesses, particularly if seafood is poorly handled or stored [7]. *C. divergens* and *L. pentosus* are often found in vacuum-packed or refrigerated seafood, producing lactic acid and other by-products that create sour flavors and texture changes, reducing product appeal [8]. Effective preservation methods are thus essential to slow microbial growth and ensure seafood safety.

Traditional preservation methods, like refrigeration and freezing, face challenges such as energy inefficiencies and texture degradation due to ice formation [9]. EOs may offer a solution by enabling higher storage temperatures and reducing dependence on low-temperature control. Studies have shown that EOs, in both solid and vapor forms, can control bacterial growth across various food products [10,11]. Volatile EOs can create a protective atmosphere within packaged foods, and EOs in the solid phase can be applied directly to food surfaces or packaging materials. However, the effects of these different application forms on EO anti-spoilage bacteria efficacy, especially in seafood, remain limited.

This study aims to investigate the antibacterial activity of citrus EOs in both solid and vapor phases against six common fish spoilage bacteria. By focusing on these bacteria, we aim to understand how EO application form impacts antibacterial properties. Furthermore, proteomic techniques will be used to explore the mechanisms of bacterial inhibition, which could improve seafood safety and shelf life through phase-specific antibacterial effects.

## 2. Results and Discussion

### 2.1. Inhibitory Effect of EOs in Solid and Vapor Phases on Growth of Spoilage Bacteria

Seven EOs were screened for their antibacterial activity in the solid and vapor phases against various bacteria. As shown in Table 1, all EOs in the solid phase inhibited the growth of Gram-negative bacteria. *V. harveyi* and *S. putrefaciens* were relatively sensitive to EOs, with diameter of inhibition zone (DIZ) ranging from 24 to 50 mm and from 18 to 61 mm, respectively. In contrast, *P. damselae* showed relatively lower sensitivity to EOs, with DIZ ranging from 11 to 20 mm. However, most EOs showed relatively lower inhibitory effects on the growth of the lactic acid bacterium *C. divergens*, and none of the EOs inhibited the growth of *L. pentosus*. Compared with other EOs, lemon and orange EOs exhibited the most remarkable inhibitory effects on bacterial growth. *L. pentosus* and *C. divergens* belong to the order *Lactobacillales*. Similarly, Man et al. reported that Gram-negative bacteria, such as *Escherichia coli*, *Klebsiella pneumoniae*, and *Pseudomonas aeruginosa*, were more sensitive to EOs than Gram-positive bacteria, such as *Staphylococcus aureus* and *Enterococcus faecalis* [12]. Lemon, lime, and orange EOs showed relatively higher antibacterial activity against most Gram-negative bacteria (Table 1), with an average inhibitory zone diameter of 28.26, 30.6, and 31.13 mm, respectively.

The antimicrobial activity of the EOs in the vapor phase was determined at a volatile concentration of 294 μL/L air per Petri dish. As shown in Table 2, lemon and orange EOs exhibited the highest overall inhibitory activity in the vapor phase against Gram-negative bacteria (Table 2). However, none of the EOs showed inhibition zone effects against *P. damselae*. For *V. parahaemolyticus*, lemon EO had the largest inhibition zone, with a mean value of 40.67 ± 1.15 mm, followed by orange EO with a mean value of 46.33 ± 5.03 mm. In addition, some EOs showed higher antibacterial activity in the vapor phase compared to the solid phase. For example, lemon EO had an inhibition zone of 74.00 ± 19.05 and 36.33 ± 0.75 mm against *S. putrefaciens* in the vapor and solid phases, respectively. Applying EOs in food preservation involves additional complexities. These include environmental factors, temperature, pH, and food system characteristics like sensory changes. Qian et al. demonstrated that the application of 200 μL of 0.25% (*v*/*v*) oregano essential oil effectively inhibited the growth of the seafood spoilage bacteria *S. putrefaciens* in an Oxford cup assay. The treatment resulted in an inhibition zone measuring 11.18 mm in diameter [13]. Luo et al. showed that oregano EO could inhibit strains of the food-borne pathogen *V. vulnificus*, with a minimum inhibitory concentration (MIC) of 0.06 to 0.15 μL/mL [14].

Meanwhile, none of the EOs could inhibit Gram-positive bacteria in the vapor phase. Based on previous reports, the peptidoglycan cell wall of lactic acid bacteria can be attached to lipoteichoic acids, polysaccharides, and proteins, which might protect lactic acid bacteria from antimicrobials [15]. The structural diversity of teichoic acids and polysaccharides could vary among bacterial species and strains, and this structural diversity often modulates susceptibility or resistance to antimicrobials. Overall, lemon and orange EOs showed the highest inhibitory activity, and their inhibitory effects at various concentrations on spoilage bacteria were further determined.

### 2.2. Effects of EOs at Various Concentrations on Spoilage Bacteria

**Figure 1:**
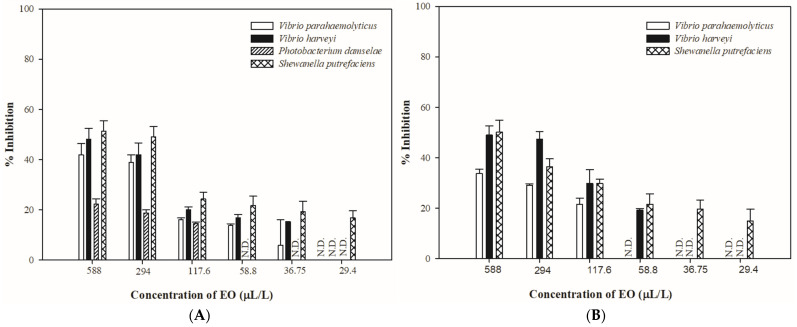
The effect of lemon essential oils in the solid phase (**A**) and vapor phase (**B**) on the test organisms at different concentrations (n = 3).

**Figure 2:**
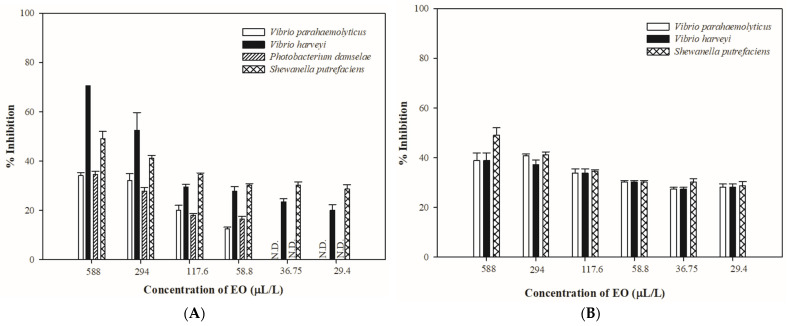
The effect of orange essential oils in the solid phase (**A**) and vapor phase (**B**) on the test organisms at different concentrations (n = 3).

Inhibition rate was measured for *V. parahaemolyticus*, *V. harveyi*, *P. damselae*, and *S. putrefaciens* to test the minimum antibacterial concentration of lemon and orange EOs in the solid and vapor phases (Figure 1 and Figure 2).

As shown in Figure 1, lemon EO exhibited antibacterial activity in a dose-dependent manner in the solid and vapor phases. *S. putrefaciens* was the most sensitive bacterium to lemon EO, whereas *P. damselae* showed the least sensitivity (Figure 1A). In the solid phase, lemon EO could retard the growth of *S. putrefaciens* at a concentration of 29.4 μL/L, with an inhibitory percentage of 16.86% ± 2.71%, but it failed to inhibit the growth of *P. damselae* at a concentration of 58.8 μL/L. Similar results were observed in the vapor phase (Figure 1B), wherein *S. putrefaciens* was inhibited at a concentration of 29.4 μL/L, with an inhibitory percentage of 14.90% ± 4.75%.

As shown in Figure 2, orange EO exhibited antibacterial activity in a dose-dependent manner in the solid and vapor phases. *V. harveyi* and *S. putrefaciens* were sensitive to orange EO, whereas *V. parahaemolyticus* and *P. damselae* were less sensitive to orange EO (Figure 2A). Orange EO could retard the growth of *V. parahaemolyticus*, *V. harveyi*, *P. damselae*, and *S. putrefaciens* at a concentration of 58.8 μL/L, with inhibitory percentages of 12.54% ± 0.67%, 27.84% ± 1.79%, 16.47% ± 1.17%, and 30.19% ± 0.67%, respectively. However, orange EO failed to inhibit the growth of *V. parahaemolyticus* and *P. damselae* at a concentration of 36.75 μL/L in the solid phase. In the vapor phase (Figure 2B), orange EO could inhibit the growth of *V. parahaemolyticus*, *V. harveyi*, and *S. putrefaciens* at all tested concentrations.

Research conducted by Qian et al. demonstrates that treating shrimp through immersion in oregano EOs (0.12%, *v*/*v*) can effectively extend their shelf life. Oregano EO has been found to inhibit the growth and proteolytic activity of *S. putrefaciens* in shrimp at both refrigerated and room temperatures. This inhibition consequently leads to a reduction in the production of volatile basic nitrogen and bioamines [13]. To date, the application of essential oils in the vapor phase for the preservation of seafood is still limited. 

### 2.3. Chemical Composition Analysis of EOs Using GC–MS

The chemical composition of EOs plays a major role in their antibacterial activity. In this study, lemon and orange EOs were analyzed via GC–MS. The identified compounds, along with their retention times and relative peak areas (%), are shown in Supplementary Table S1, Table S2, Figure S1 and Figure S2, respectively. A total of 24 compounds were identified in lemon EO, accounting for 99.7% of the total volatile components. Limonene (64.92%) was the dominant compound, followed by *β*-pinene (12.42%), *γ*-terpinene (8.94%), *α*-pinene (2.09%), sabinene (2.09%), *β*-myrcene (1.37%), and p-cymene (2.02%), which together accounted for >1% of the total components. The chemical composition of lemon EO (Supplementary Table S1) showed similarities to a previously reported analysis of California lemon EO [16]. However, significant differences were observed when comparing EOs originating from Italy and Turkey. According to Poiana et al. and Gök et al., lemon EOs from Italy had higher levels of *β*-pinene and *γ*-terpinene, while those from Turkey had relatively higher limonene and lower *β*-pinene content, compared with the cold-pressed lemon EO used in this study [17,18].

In orange EO, 22 chemical compounds were quantified, accounting for 99.8% of the identified compounds. Limonene (94.66%) was the primary component in orange EO (Supplementary Table S2), a finding consistent with previous studies, which also reported limonene to be the most abundant compound in orange EO, regardless of the extraction method [19,20]. Other major components (i.e., >0.4%) included *β*-myrcene (1.76%), *α*-pinene (0.65%), linalool (0.44%), and valencene (0.45%). Based on the GC–MS results, monoterpenes were the predominant class in lemon and orange EOs, while sesquiterpenes, hydrocarbons, alcohols, aldehydes, and esters were present in relatively lower concentrations. The major constituent, limonene, is known for its moderate citrusy and peel-like odor, making it a key ingredient in citrus-flavored products. The other main components in lemon EO (peak no. 2, 4, 5, 6, 10, and 14) contribute citrus, mandarin, medicinal, pine, petroleum, mango, and woody notes. In contrast, the major components in orange EO (peak no. 3, 6, 10, 13, and 24) impart citrus, floral, herbal, lavender, pine, mango, orange, and woody notes.

### 2.4. Effects of Lemon and Orange EOs on Membrane Integrity of Spoilage Bacteria

Proteins and nucleic acids are crucial macromolecules for cellular structure and genetic information. The effects of lemon and orange EOs on the membrane integrity of *V. parahaemolyticus*, *V. harveyi*, *P. damselae*, and *S. putrefaciens* were evaluated by monitoring changes in extracellular nucleic acid (A260) and protein concentration using protein assays.

In preparation for an examination of the impact of EOs on the membrane integrity of spoilage bacteria, we first determined the MICs of lemon and orange EOs against various seafood-associated bacteria (Supplementary Table S3). This determination was conducted using the broth dilution method. The results revealed that the MICs of lemon EO against *V. parahaemolyticus*, *V. harveyi*, *P. damselae*, and *S. putrefaciens* were 2352, 4704, 2352, and 588 μL/L, respectively. For orange EO, the MICs against the same bacteria were 1176, 2352, 588, and 588 μL/L, respectively.

For *V. parahaemolyticus*, after treatment with lemon EO at 1/4 MIC and the MIC, the absorbance at 260 nm increased in a dose-dependent manner from 0.06 ± 0.01 to 0.09 ± 0.02 and 0.24 ± 0.01, respectively. The increase in A260 indicated the leakage of cellular constituents, such as nucleic acids, suggesting that lemon EO treatment destabilizes or damages the cell membrane of *V. parahaemolyticus*. Similar phenomena were observed for other bacteria (*V. harveyi*, *P. damselae*, and *S. putrefaciens*) treated with lemon EO. The absorbance at 260 nm for *V. parahaemolyticus* treated with orange EO at 1/4 MIC and the MIC also increased in a dose-dependent manner from 0.06 ± 0.00 to 0.07 ± 0.05 and 0.14 ± 0.01, respectively. Similar phenomena were observed for other bacteria (*V. harveyi*, *P. damselae*, and *S. putrefaciens*) treated with orange EO. These results indicate that treatment with lemon and orange EOs could increase extracellular protein concentrations, and the tested microorganisms may have varying susceptibility to EOs (Table 3). 

Based on previous studies, the bioactive compounds in EOs are known to damage various cell structures, such as cell walls and membranes, and disrupt the proton motive force [21]. Our data suggest that treatment with lemon and orange EOs increases extracellular nucleic acids and proteins, indicating cell membrane destabilization. However, further research is required to confirm the mechanisms of action.

EOs, as food preservatives, offer significant environmental benefits and are more sustainable than traditional chemical preservatives. While traditional chemical preservatives (such as benzoic acid and sorbic acid) effectively inhibit microbial growth, they often persist in water or soil after use [22]. Their resistance to complete biodegradation can result in long-term environmental pollution and toxic effects on aquatic organisms and microbial communities. In contrast, EOs are derived from renewable plant resources, such as lemons and oranges. Their extraction processes are relatively environmentally friendly, and their waste degrades without causing persistent pollution. The primary active components of EOs (such as limonene and citral) decompose easily in nature, posing no long-term risks to aquatic or soil ecosystems.

### 2.5. Proteome Analysis

Proteomic technology was used for a comprehensive analysis to investigate the effects of EOs on *V. parahaemolyticus*. Differentially expressed proteins (DEPs) were defined as proteins showing at least a 20% change in expression levels with *p*-values < 0.05.

**Figure 3:**
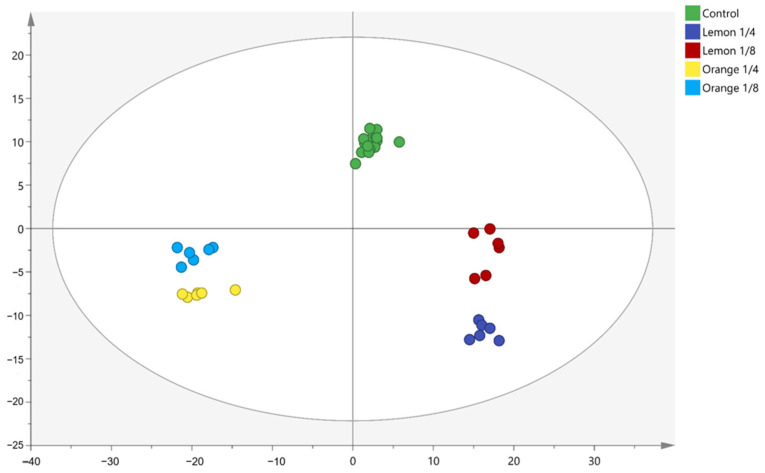
PCA plot of *V. parahaemolyticus* proteins treated with different concentrations of lemon EO and orange EO.

**Figure 4:**
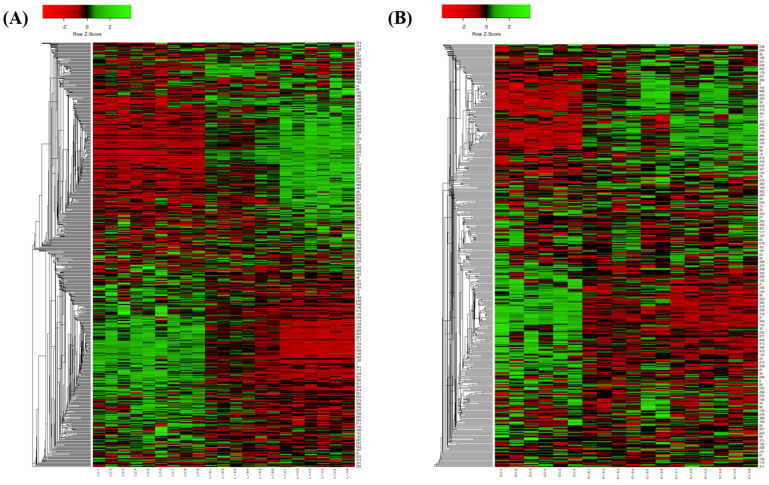
Heatmap of *V. parahaemolyticus* proteins treated with different concentrations of (**A**) lemon EO and (**B**) orange EO.

Principal component analysis (PCA) revealed widely dispersed data points between the EO-treated and control groups. The heatmap indicated significant differences in most detected proteins between the two groups, highlighting the impact of EOs on *V. parahaemolyticus* (Figure 3 and Figure 4).

**Figure 5:**
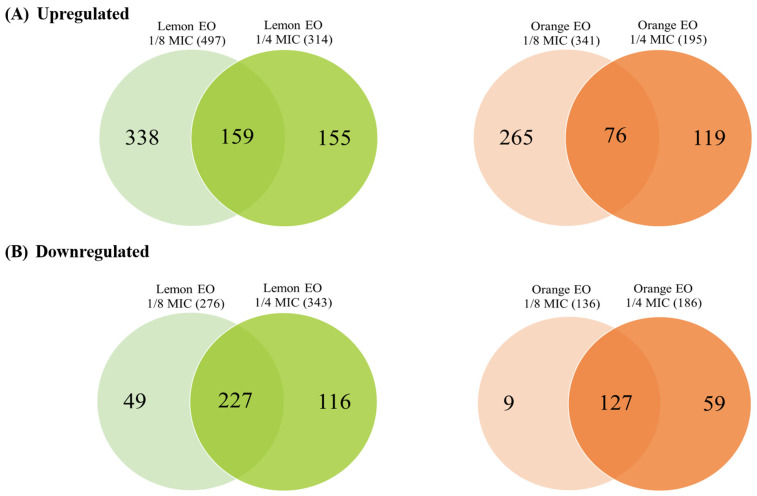
Venn diagrams of protein expression (**A**) upregulation and (**B**) downregulation in *V. parahaemolyticus* treated with lemon and orange EOs at different concentrations.

**Figure 6:**
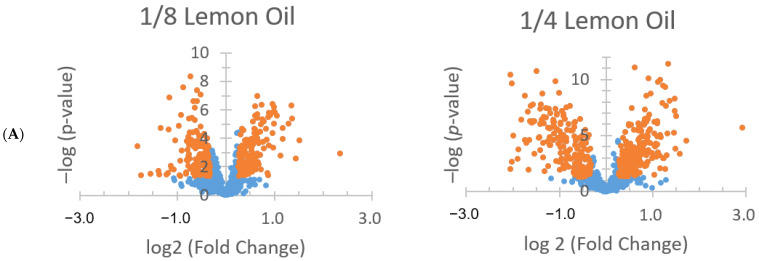

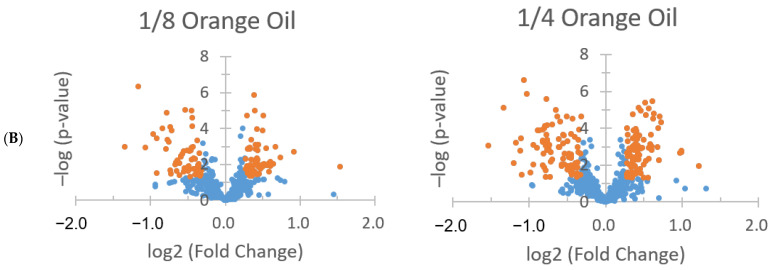
Volcano plots of protein expression changes in *V. parahaemolyticus* treated with different concentrations of lemon (**A**) and orange (**B**) EO. All the dots represent a protein, and each orange dot represents a DPE.

Figure 5 shows the upregulated and downregulated proteins for both EOs. Lemon EO upregulated 497 proteins and downregulated 276 proteins at 1/8 MIC, whereas at a 1/4 MIC, it upregulated 314 proteins and downregulated 343 proteins. Orange EO upregulated 341 proteins and downregulated 136 proteins at 1/8 MIC, and at 1/4 MIC, it upregulated 195 proteins and downregulated 186 proteins. The changes in protein expression levels were also observed through volcano plots. Figure 6 presents the volcano plots of protein expression for *V. parahaemolyticus* treated with two concentrations of EOs, with orange dots representing DEPs. In the lemon EO group, 1/4 MIC resulted in a higher number of DEPs than 1/8 MIC. A similar pattern was observed in orange EO, indicating that more proteins were significantly upregulated or downregulated at 1/4 MIC. Lemon EO showed a significantly larger number of DEPs at both concentrations compared with orange EO.

PANTHER GO analysis was also performed on proteins with dose-dependent expression to determine the significantly affected pathways. The enrichment value represents the degree of impact on the pathway. Under lemon EO treatment, proton-transporting ATP synthase activity and transmembrane signaling receptor activity showed a high degree of enrichment in molecular functions, suggesting that lemon EO enhances ATP synthesis and cell signaling. In biological processes, increases in chemotaxis and proton motive force-driven ATP synthesis were observed, indicating improved bacterial motility and energy metabolism. Proteins related to ATP synthesis were significantly upregulated in cellular components, protein classes, and biological pathways. In contrast, proteins associated with protein translation and glycolysis pathways were significantly downregulated. Notably, proteins related to antioxidant and oxidoreductase activities were also downregulated (Table 4). In the group treated with orange EO, significantly upregulated proteins were associated with ATPase activity, ATP synthesis/binding, signal peptides, and oxidoreductase activity. Conversely, GTPase activity, hexose, leucine biosynthesis, and the glycolysis pathway were significantly downregulated (Table 5).

Terpenoids in EOs could increase the level of reactive oxygen species (ROS) in bacteria, leading to elevated oxidative stress. Accumulation of ROS can damage DNA, proteins, or cell membranes, thereby causing bacterial death [23,24]. Our proteomic analysis confirms that treatment with lemon or orange EO induces oxidative reactions within cells. In response to this stress, various proteins related to DNA repair show significant upregulation. Among these proteins, ligase, MutS, and GyrA require ATP for the repair process [25,26]. In addition, the protease ClpX showed significant upregulation. Research indicates that under stress conditions, ClpX facilitates the degradation of misfolded proteins, a process also requiring ATP [27,28]. These findings align with our proteomic results, which show significant upregulation of proton transport ATP synthase and ATP synthesis pathways in cells treated with both EOs. Furthermore, protein synthesis pathways were downregulated in both EO treatment groups, with some differences observed. Lemon EO significantly downregulated proteins related to translation factors, suggesting a strong inhibitory effect on protein synthesis.

Pathways associated with antioxidant activity were downregulated in the lemon EO group but upregulated in the orange EO group. In particular, the expression levels of five antioxidant proteins (SodF, Bcp, TrxB, YfeX, and Gor) were notably reduced in the lemon EO group, indicating that lemon EO decreases bacterial capacity for free radical scavenging, exposing cells to increased oxidative stress. This increased oxidative stress may explain the stronger antibacterial activity of lemon EO compared with orange EO. The antibacterial effect of orange EO may be attributed to its impact on GTPase activity pathways, as three proteins (FtsZ, ObgE, and BipA) related to GTPase activity were downregulated. FtsZ is a tubulin homolog, and ObgE is a GTPase; both are crucial for bacterial cell division, with FtsZ being a major cytoskeletal protein and GTPase providing the energy required for FtsZ assembly. The inhibition of FtsZ or GTPase activity disrupts bacterial cell division, leading to cell death [29]. Proteomic analysis offers a comprehensive understanding of the effects of lemon and orange EOs on bacteria, providing insights into their antibacterial mechanisms from various perspectives. These results are valuable for exploring the use of EOs as natural antibacterial agents.

While this study provides significant insights into the antibacterial properties and mechanisms of citrus EOs, it is important to acknowledge that practical applications may present additional complexities. These include environmental factors and food system dynamics that could influence EOs’ effectiveness. For example, the antibacterial efficacy of these essential oils in seafood preservation may vary depending on storage conditions such as temperature and pH. Notwithstanding these limitations, our research establishes a robust foundation for future investigations. We anticipate that subsequent studies will build upon this work, evaluating the performance of EOs under real-world conditions and ensuring their safety across diverse applications.

## 3. Materials and Methods

### 3.1. Strains and EOs

Four strains were used in this study: *P. damselae* subsp. *damselae* BCRC 17130, *S. putrefaciens* BCRC 10596, *C. divergens* BCRC 14042, and *L*. *pentosus* BCRC 80017, all purchased from the Bioresources Collection and Research Center (BCRC) in Hsinchu City. *V. parahaemolyticus* and *V. harveyi* were isolated from diseased fish in a previous study [30]. Following BCRC instructions, the bacteria were incubated in Tryptic Soy Broth (TSB), MRS broth, or Mueller–Hinton (MH) broth (Formedium, Norfolk, UK) at 30 °C or 26 °C. 

Seven citrus EOs were used: folded lemon essential oil (lemon EO) (Citromax Inc., Carlstadt, NJ, USA), bergamot oil (Kris Aromatics Ltd., Hertfordshire, UK), orange oil cp. (B.W.I Chemicals, Cranbury, NJ, USA), lime oil expressed (De Monchy Aromatics Ltd., Poole, UK), mandarin oil red (Kris Aromatics Ltd., Hertfordshire, UK), mandarin oil green (Kris Aromatics Ltd., Hertfordshire, UK), and grapefruit essential oil (grapefruit EO) (Misitano & Stracuzzi S.p.A., Messina, Italy). 

### 3.2. Solid/Vapor Phase Diffusion Test

The solid/vapor phase diffusion test was conducted according to the method of Lopez et al. with modifications [31]. A single colony was inoculated into 10 mL of a fresh medium and incubated to an OD600 of 0.6–0.8. The culture was diluted to 10^5^ CFU/mL and inoculated onto suitable agar culture media. Different concentrations of EO were added at 10 μL to 8 mm paper disks, which were placed in the center of the agar or on the cover of the dish, and the dishes were sealed with paraffin film. Disks soaked in ethanol were used as the control. After incubation for 12–16 h, DIZ was measured.

The concentration of the tested EO was calculated using the following formula:

$$EO\;concentration\;(\mu L/L\;air)=\frac{V}{\pi r^{2}h}$$

V: the volume of EO added (μL); π: 3.14; r: the radius of the Petri dish (cm); h: the height of the reserved Petri dish (cm). Various concentrations of EO were prepared using 95% ethanol as the diluent, and the experimental EO concentrations of 588–29.4 μL/L air were prepared for the test.

### 3.3. MIC Assay

The MIC of various EOs against food spoilage bacteria was determined using a modified broth dilution method, as described by Lu et al. [32]. In brief, the MIC was assessed by cultivating bacteria in their logarithmic growth phase (OD600 = 0.6–0.8) and suspending them in MH broth to achieve a density of 10^5^ CFU/mL. DMSO was added to the EO stock to achieve a final DMSO concentration of 5% (*v*/*v*). Each EO underwent a two-fold serial dilution in 100 μL of MH broth within a U-bottomed 96-well plate. Subsequently, 100 μL of bacterial suspension was added to each well, resulting in a final volume of 200 μL. The plates were incubated at 30 °C (for *V. parahaemolyticus* and *V. harveyi*) or 26 °C (for *P. damselae*, *S. putrefaciens*) for 16 to 18 h, after which bacterial growth was evaluated to determine the MICs (μL/L total solution). 

### 3.4. Material (DNA and Protein) Leakage Test

The material leakage test was conducted according to the method of Zhang et al. with modifications [33]. A single colony was inoculated into 100 mL of MH broth or MH broth containing 2% NaCl and incubated overnight. The bacterium was then centrifuged at 6000 rpm, and the pellet was washed three times with equal volumes of PBS buffer (0.1 M, pH 7.4). The 100 mL PBS suspension was transferred into new centrifuge tubes, with 20 mL per tube. DMSO was added to the EO stock to achieve a final DMSO concentration of 5% (*v*/*v*). The MIC and 1/4 MIC of EO were mixed with the bacterial suspension, while a sample without EO was used as the control. All samples were incubated at the optimal temperature with shaking at 150 rpm. After 6 h, the samples were centrifuged at 6000 rpm, and the supernatant was measured for OD 260 nm. The protein concentration in each sample was measured using the BCA Protein Assay (Thermo, Waltham, MA, USA).

### 3.5. Gas Chromatography–Mass Spectrometry (GC-MS)

The volatile compounds in the EOs were analyzed by the Agilent GC-MS system. The procedure and equipment were described in Lin et al. [11]. GC-MS was used to analyze the components of the two EOs with the best antibacterial effects. This part of the experiment was conducted with assistance from the analysis by Guang Fulin Co., Ltd. (Taoyuan, Taiwan). The gas chromatography part was separated by a VF-5 ms capillary column (length 60 m, inner diameter 0.32 mm, film thickness 0.25 μm) with the Agilent GC-MS system (Agilent Technologies, Santa Clara, CA, USA). The mobile phase was helium (helium, He) at 21 lbf. Under constant pressure (pounds per square inch, psi), the sample was injected in a 20:1 split mode. The EO sample was diluted 1:30 (*v*/*v*) with hexane, and the sample injection volume was 0.1 μL. The initial temperature of the column was 50 °C for 5 min, increasing first at a rate of 5 °C per minute to 105 °C, then at a rate of 1 °C per minute to 120 °C, and then at a rate of 5 °C per minute. The rate was raised to 202 °C and finally was raised to 275 °C at a rate of 10 °C per minute for 15.3 min. The temperature of the injector and ion source was 250 °C and 230 °C, respectively. The Agilent 5973 mass spectrometer system (Agilent Technologies, Santa Clara, CA, USA) ionized the sample at 70 eV, and the scan range of the mass spectrometer was 20–400 *m*/*z*.

The mass spectrometry data of volatile compounds and the standards provided in the NIST 08 database were used to identify the composition of EOs.

### 3.6. Protein Extraction, Label-Free Proteomics Analysis, and Pathway Analysis

Protein sample extraction and preparation were conducted according to the method of Zhang et al. and Hsu et al. with modifications [33,34]. A single colony was inoculated into 100 mL of MH broth or MH broth containing 2% NaCl and incubated overnight. The bacterium was then centrifuged at 6000 rpm, and the pellet was washed three times with equal volumes of PBS buffer (0.1 M, pH 7.4). The 100 mL PBS suspension was transferred into new centrifuge tubes, with 20 mL per tube. DMSO was added to the EO stock to achieve a final DMSO concentration of 5% (*v*/*v*). The 1/4 and 1/8 MICs of EO were mixed with the bacterial suspension, while a sample without EO was used as the control group. All samples were incubated at the optimal temperature with shaking at 150 rpm for 1.5 h.

The bacterium was then centrifuged at 6000 rpm, and the pellet was washed three times with PBS buffer. The pellet was resuspended in 5× volume of PBS buffer and sonicated (30% power, 10 s on, 10 s off, for a total of 10 min) to lyse the cells. The supernatant was separated by centrifugation at 6000 rpm for 10 min, and the protein in the supernatant was precipitated with iced acetone at −20 °C overnight. After protein extraction, the acetone was removed by centrifugation at 8000 rpm for 10 min. The pellet was resuspended in 5× volume of 100 mM TEAB buffer, and the concentration was measured using the BCA Protein Assay (Thermo, Waltham, MA, USA).

For further processing, 150 μg of protein was transferred into a new Eppendorf tube, and 100 mM TEAB buffer was added to reach a total volume of 100 μL. The protein solution was reacted with 200 mM DTT at 55 °C for 30 min, followed by the addition of 1 M IAA, which was allowed to react at room temperature for 1 h. Then, 10–20 μL of trypsin solution was added, and the mixture was gently vortexed and centrifuged. To ensure complete protein digestion, the sample was placed on a thermomixer at 25 °C overnight.

The sample preparation involved desalting using a Waters SPE Oasis HLB column. The column was first activated with 1 mL of acetonitrile (ACN) and then equilibrated with 1 mL of double-distilled water. After loading the sample, the column was washed with double-distilled water and the peptides were eluted twice with 70% ACN. The eluted peptides were then dried using a Speed-Vac and prepared for mass spectrometry analysis. The dried samples were stored at room temperature prior to analysis.

The LC-MS/MS data were acquired using an Orbitrap Fusion mass spectrometer (Thermo, Waltham, MA, USA) coupled with an EASY-nLC 1200 liquid chromatography system (Thermo, Waltham, MA, USA). The peptides were separated on an EASY-Spray HPLC column (75 µm I.D. × 150 mm, 3 µm, 100 Å) using a binary solvent system: mobile phase A was 0.1% formic acid in water, and mobile phase B was 0.1% formic acid in 80% acetonitrile. The LC gradient was set to 2% buffer B for 2 min, followed by a linear increase to 40% buffer B over 100 min, at a flow rate of 500 nL/min. The electrospray ionization source was operated at 1.8 kV, and the capillary temperature was maintained at 275 °C. Full MS survey scans were performed in the mass range of *m*/*z* 320–1600, with an automatic gain control (AGC) target of 5 × 10^5^ and a resolution of 120,000 (at *m*/*z* 200) with a maximum injection time of 50 ms.

The MS raw data were analyzed using MaxQuant (ver. 1.6.14) software for protein quantitation. The database search parameters included a precursor mass tolerance of 10 ppm, a fragment ion mass tolerance of 0.6 Da, and a maximum of 3 allowed enzyme missed cleavages. The identified peptides were filtered to maintain a false discovery rate (FDR) of 5% or less. The quantified protein abundances were then subjected to PANTHER (http://www.pantherdb.org, accesed on 30 April 2024) [35] for cell signaling pathway and biological function analysis. A significance threshold of *p* < 0.05 was applied for the PANTHER analysis.

### 3.7. Statistical Analysis

All experiments were conducted in triplicate. Data were expressed as mean ± SD and statistically analyzed using IBM SPSS Statistics 23.0 (IBM, Armonk, NY, USA). Statistically significant differences between the samples were determined by one-way analysis of variance (ANOVA) and Tukey’s honestly significant difference (HSD) test, where *p* < 0.05 indicated significant differences.

## 4. Conclusions

Our study demonstrated that citrus EOs have effective antibacterial properties against Gram-negative spoilage bacteria, with lemon and orange EOs showing dose-dependent inhibition in both solid- and vapor-phase applications. Proteomic analysis revealed distinct mechanisms: lemon EO affected bacterial antioxidant defenses, while orange EO disrupted cell division. These findings offer valuable insights into the potential of different EO application forms in controlling spoilage bacteria. Although challenges remain, such as optimizing EO concentrations and ensuring their stability, this study provides a foundation for exploring EOs as a natural option to enhance food safety.

## Figures and Tables

**Table 1:** The solid-phase antibacterial ability of essential oils against spoilage bacteria in seafood.

Microorganisms	DIZ * (mm)						
	Lemon Oil Cp	Lime Oil Expressed	Orange Essence Oil	Bergamot Oil	Mandarin Oil Red	Mandarin Oil Green	Grapefruit Essence 3-Fdd
Gram-negative							
*V. parahaemolyticus*	26.67 ± 1.15 ^bcBC^	28.00 ± 2.00 ^bcB^	32.00 ± 2.00 ^bA^	28.00 ± 2.00 ^bB^	24.00 ± 2.00 ^abBC^	28.00 ± 2.00 ^bB^	30.00 ± 2.00 ^bA^
*V. harveyi*	50.33 ± 3.51 ^aA^	42.67 ± 2.31 ^abB^	45.67 ± 1.53 ^aAB^	42.00 ± 4.00 ^aB^	24.00 ± 5.29 ^abC^	45.67 ± 2.89 ^aAB^	46.00 ± 5.29 ^aAB^
*P. damselae*	12.67 ± 0.58 ^cC^	13.67 ± 0.058 ^bcC^	20.67 ± 1.15 ^cA^	15.50 ± 1.32 ^cB^	11.00 ± 0.00 ^bD^	12.50 ± 0.87 ^cC^	15.17 ± 0.76 ^cB^
*S. putrefaciens*	37.33 ± 1.12 ^abAB^	61.66 ± 2.03 ^aA^	42.33 ± 0.64 ^aAB^	26.33 ± 0.47 ^bB^	60.50 ± 3.46 ^aA^	26.66 ± 0.51 ^bB^	18.66 ± 0.15 ^cB^
Gram-positive							
*L. pentosus*	ND	ND	ND	ND	ND	ND	ND
*C. divergens*	14.33 ± 1.15 ^cB^	7.00 ± 0.71 ^cB^	15.00 ± 1.00 ^cA^	ND	ND	10.67 ± 1.15 ^cB^	14.33 ± 2.52 ^cA^

* DIZ, diameter of inhibition zone, including 8 mm diameter of EO disk. ND = non-detected. Different capital letters in the same row show significant differences between the bacteria treated with various EOs. Different lower-case letters in the same column show significant differences between the different bacteria treated with the same EO.

**Table 2:** The vapor-phase antibacterial ability of essential oils against spoilage bacteria in seafood.

Microorganisms	DIZ * (mm)						
	Lemon Oil Cp	Lime Oil Expressed	Orange Essence Oil	Bergamot Oil	Mandarin Oil Red	Mandarin Oil Green	Grapefruit Essence 3-Fdd
Gram-negative							
*V. parahaemolyticus*	40.67 ± 1.15 ^aAB^	36.00 ± 3.46 ^abBC^	46.33 ± 3.79 ^aA^	18.00 ± 5.29 ^aD^	32.67 ± 3.06 ^bC^	37.33 ± 2.31 ^bBC^	36.00 ± 4.00 ^aBC^
*V. harveyi*	45.33 ± 4.16 ^aA^	42.00 ± 9.17 ^aA^	46.67 ± 5.03 ^aA^	24.67 ± 5.03 ^aB^	44.00 ± 4.00 ^aA^	43.33 ± 3.06 ^aA^	52.67 ± 9.87 ^aA^
*P. damselae*	ND	ND	ND	ND	ND	ND	ND
*S. putrefaciens*	68.67 ± 28.29 ^aA^	19.50 ± 3.54 ^bB^	48.33 ± 32.52 ^aAB^	29.67 ± 7.37 ^aAB^	12.67 ± 1.15 ^cB^	ND	46.33 ± 33.62 ^aAB^
Gram-positive							
*L. pentosus*	ND	ND	ND	ND	ND	ND	ND
*C. divergens*	ND	ND	ND	ND	ND	ND	ND

* DIZ, diameter of inhibition zone, including 8mm diameter of EO disk. ND, non-detected. Different capital letters in the same row show significant differences between the bacteria treated with various EOs. Different lower-case letters in the same column show significant differences between the different bacteria treated with the same EO.

**Table 3:** Measurements of nucleic acids and proteins from bacteria treated with lemon and orange EO.

	Microorganisms			
	*V. parahaemolyticus*	*V. harveyi*	*P. damselae*	*S. putrefaciens*
	Absorbance at 260 nm			
Lemon EO				
Control (cell not treated)	0.06 ± 0.01 ^c^	0.06 ± 0.00 ^b^	0.02 ± 0.00 ^c^	0.08 ± 0.01 ^b^
1/4 MIC	0.09 ± 0.06 ^b^	0.70 ± 0.05 ^a^	0.75 ± 0.02 ^b^	0.45 ± 0.03 ^a^
MIC	0.24 ± 0.01 ^a^	0.77 ± 0.05 ^a^	0.79 ± 0.02 ^a^	0.45 ± 0.03 ^a^
Orange EO				
Control (cell not treated)	0.06 ± 0.01 ^c^	0.05 ± 0.00 ^c^	0.07 ± 0.04 ^c^	0.08 ± 0.01 ^c^
1/4 MIC	0.09 ± 0.05 ^b^	0.86 ± 0.04 ^b^	0.65 ± 0.01 ^b^	0.28 ± 0.00 ^b^
MIC	0.15 ± 0.01 ^a^	0.95 ± 0.06 ^a^	0.66 ± 0.02 ^a^	0.36 ± 0.02 ^a^
	Protein concentration (μg/mL)			
Lemon EO				
Control (cell not treated)	64.65 ± 4.78 ^c^	2.00 ± 1.11 ^b^	38.40 ± 3.63 ^c^	27.33 ± 9.76 ^b^
1/4 MIC	109.61 ± 6.5 ^b^	73.70 ± 7.63 ^a^	157.97 ± 8.71 ^a^	126.95 ± 6.49 ^a^
MIC	208.78 ± 21.02 ^a^	73.79 ± 4.00 ^a^	132.25 ± 5.95 ^b^	127.71 ± 26.17 ^a^
Orange EO				
Control (cell not treated)	64.65 ± 4.78 ^c^	2.00 ± 1.11 ^b^	31.80 ± 0.00 ^c^	19.51 ± 7.46 ^b^
1/4 MIC	97.05 ± 16.15 ^b^	89.40 ± 1.48 ^a^	100.60 ± 1.66 ^a^	53.17 ± 1.21 ^a^
MIC	217.60 ± 11.57 ^a^	89.87 ± 4.07 ^a^	92.25 ± 2.28 ^b^	53.38 ± 5.90 ^a^

Different lower-case letters in the block show significant differences between the bacteria treated with different concentrations of EO.

**Table 4:** PANTHER GO enrichment analysis of dose-dependent upregulated and downregulated proteins in *V. parahaemolyticus* treated with lemon EO.

PANTHER GO Enrichment Analysis	Lemon EO Dose-Dependent Protein		
	#	Fold Enrichment	MF/BP/CC/Protein Class Classification
upregulated			
Molecular Function			
proton-transporting ATP synthase activity, rotational mechanism	5	17.46	transporter activity
transmembrane signaling receptor activity	4	16.77	molecular transducer activity
ATP binding	6	5.99	binding
ATP-dependent activity	8	3.22	ATP-dependent activity
Biological Process			
chemotaxis	5	14.97	locomotion/response to stimulus
proton motive force-driven ATP synthesis	3	12.57	metabolic process/cellular process
Cellular Component			
proton-transporting ATP synthase complex	4	16.77	protein-containing complex/cellular anatomical entity
cytoplasm	41	1.63	cellular anatomical entity
Protein Class			
ATP synthase	6	13.97	transporter
translation elongation factor	5	9.53	translational protein
aminoacyl-tRNA synthetase	6	5.24	translational protein
reductase	9	3.2	metabolite interconversion enzyme
DNA metabolism protein	11	2.68	DNA metabolism protein
Pathways			
ATP synthesis	3	15.72	
de novo purine biosynthesis	7	6.38	
downregulated			
Molecular Function			
translation initiation factor activity	2	16.24	binding/translation regulator activity
translation elongation factor activity	4	12.99	binding/translation regulator activity
ribosome binding	8	10.83	binding
antioxidant activity	5	6.77	antioxidant activity
oxidoreductase activity, acting on the CH-CH group of donors	4	5.91	catalytic activity
structural constituent of ribosome	9	3.75	structural molecule activity
transition metal ion binding	9	3.4	binding
DNA binding	16	2.45	binding
Biological Process			
translational elongation	3	12.18	metabolic process/cellular process
negative regulation of translation	3	9.74	biological regulation
hexose biosynthetic process	4	9.28	metabolic process
phosphate-containing compound metabolic process	15	2.41	metabolic process/cellular process
heterocycle biosynthetic process	16	2.41	metabolic process/cellular process
organophosphate metabolic process	13	2.4	metabolic process/cellular process
aromatic compound biosynthetic process	15	2.32	metabolic process/cellular process
organic cyclic compound biosynthetic process	16	2.2	metabolic process
amino acid metabolic process	16	2.15	metabolic process/cellular process
Cellular Component			
cytosol	52	2.37	cellular anatomical entity
plasma membrane	5	0.29	cellular anatomical entity
Protein Class			
translation release factor	3	9.74	translational protein
translation initiation factor	3	8.12	translational protein
translation elongation factor	5	7.38	translational protein
aminoacyl-tRNA synthetase	6	4.06	translational protein
ribosomal protein	13	3.41	translational protein
metabolite interconversion enzyme	103	1.49	metabolite interconversion enzyme
transporter	7	0.27	transporter
Pathways			
glycolysis	4	9.28	

#, number of proteins.

**Table 5:** PANTHER GO enrichment analysis of dose-dependent upregulated and downregulated proteins in *V. parahaemolyticus* treated with orange EO.

PANTHER GO Enrichment Analysis	Orange EO Dose-Dependent Protein		
	#	Fold Enrichment	MF/BP/CC/Protein Class Classification
upregulated			
Molecular Function			
proton-transporting ATP synthase activity, rotational mechanism	4	21.42	transporter activity/catalytic activity
ATP binding	4	6.12	binding
oxidoreductase activity, acting on the CH-OH group of donors, NAD or NADP as acceptor	6	5.21	catalytic activity
peptidase activity	5	4.72	catalytic activity
RNA binding	5	4.59	binding
transferase activity	19	2.06	catalytic activity
Biological Process			
signal peptide processing	2	21.42	metabolic process
proton motive force-driven ATP synthesis	3	19.27	metabolic process/cellular process
amide biosynthetic process	7	3.46	metabolic process/cellular process
carboxylic acid biosynthetic process	9	3.21	metabolic process/cellular process
amino acid metabolic process	10	2.65	metabolic process/cellular process
Protein Class			
ATP synthase	4	14.28	transporter
ribosomal protein	7	3.63	translational protein
protease	10	3.49	protein-modifying enzyme
transferase	20	2.09	metabolite interconversion enzyme
Pathways			
ATP synthesis	3	24.09	
downregulated			
Molecular Function			
GTPase activity	3	17.19	catalytic activity
translation factor activity, RNA binding	3	14.74	binding/translation regulator activity
nucleotide binding	8	4.3	binding
transferase activity	19	2.2	catalytic activity
Biological Process			
leucine biosynthetic process	2	22.92	metabolic process/cellular process
hexose biosynthetic process	3	14.74	metabolic process
glucose metabolic process	4	10.58	metabolic process
purine ribonucleotide metabolic process	4	6.88	metabolic process/cellular process
organophosphate biosynthetic process	7	4.15	metabolic process/cellular process
Cellular Component			
cytosol	29	2.79	cellular anatomical entity
Protein Class			
translation factor	5	7.16	translational protein
ribosomal protein	7	3.88	translational protein
transferase	19	2.12	metabolite interconversion enzyme
Pathways			
glycolysis	3	14.74	

#, number of proteins.

## Data Availability

The original contributions presented in this study are included in the article/Supplementary Material. Further inquiries can be directed to the corresponding author(s).

## Supplementary Materials

The following supporting information can be downloaded at https://www.mdpi.com/article/10.3390/antibiotics13121137/s1, Figure S1: GC-MS chromatograms of lemon EO; Figure S2: GC-MS chromatograms of orange EO; Table S1: Principal compounds identified in lemon EO through GC-MS analysis; Table S2: Principal compounds identified in orange EO through GC-MS analysis; Table S3: The MICs of lemon and orange EOs against various seafood-associated bacteria.
