# Supplementary material for: Solid- and Vapor-Phase Antibacterial Activities and Mechanisms of Essential Oils Against Fish Spoilage Bacteria
Source: Antibiotics (Basel). 2024 Nov 26;13(12):1137. doi: 10.3390/antibiotics13121137 (PMC11672770; doi:10.3390/antibiotics13121137)
Supplement: Supplementary file 1 [file antibiotics-13-01137-s001.zip › antibiotics-3316943-supplementary.pdf]

Supplementary Materials

Table S1. Principal compounds identified in lemon EO through GC-MS analysis.

No.	Compound	Retention Time (min)	Peak Area (%)
1	α-Thujene	9.2	0.39
2	α-Pinene	9.49	1.83
3	Camphene	10	0.06
4	Sabenene (Sabinene)	10.64	1.95
5	β-Pinene	10.92	11.77
6	β-Myrcene	11	1.53
7	Octanal	11.47	0.06
8	α-Phellandrene	11.64	0.05
9	α-Terpinene	11.96	0.18
10	D-limonene+ϱ-Cymene	12.67	67.85
11	Ocimene	12.8	0.1
12	γ-Terpinene	13.37	8.69
13	Terpinolene	14.14	0.36
14	Linalool	14.51	0.09
15	Nonanal	14.65	0.08
16	Citronellal	16.15	0.08
17	4-Terpineol	17.25	0.04
18	α-Terpineol	17.7	0.14
19	Decanal	17.81	0.05
20	Nerol	18.47	0.04
21	Citronellol	18.67	0.01
22	Neral	18.95	0.75
23	Geraniol	18.67	0.01
24	Geranial	19.84	1.23
25	Perillaldehyde	20.26	0.02
26	Undecanal	20.88	0.02
27	Citronellyl acetate	22.01	0.03
28	Neryl acetate	22.31	0.42
29	Geranyl acetate	22.86	0.27
30	β-Caryophyllene	24.43	0.2
31	Bergamontene	24.58	0.37
32	Farnesene	24.93	0.03
33	Valencene	26.49	0.04
34	β-Bisabolene	26.59	0.58
35	α-Bisabolol	31	0.02
36	5,7-Dimethyl coumarin	36.8	0.04

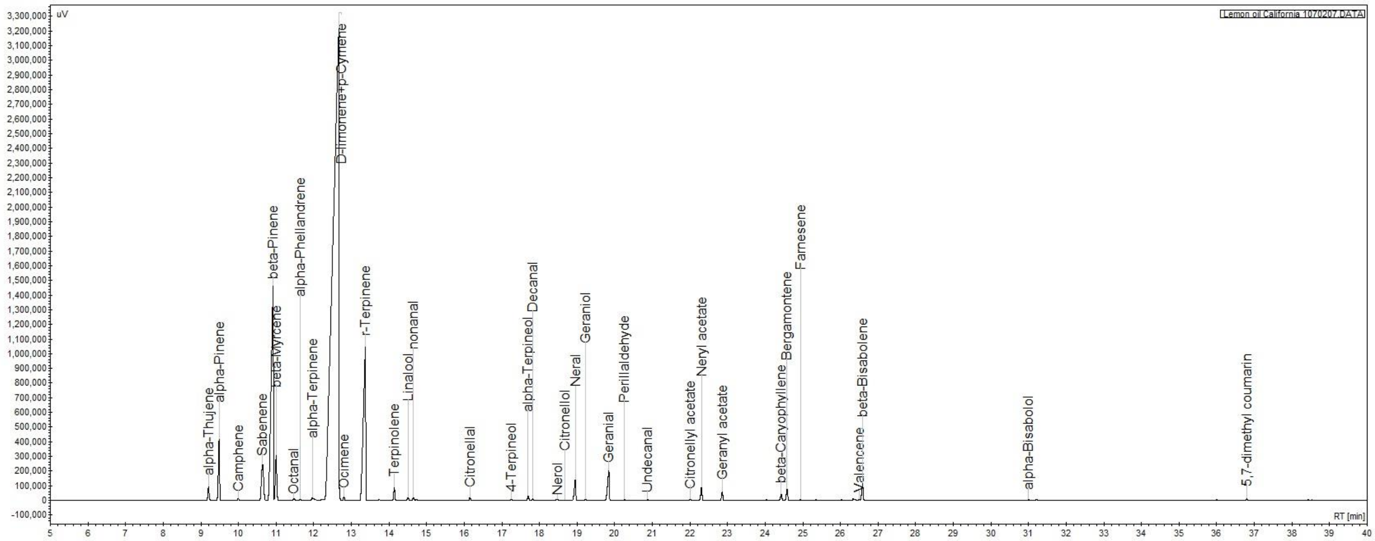

Figure S1. GC-MS chromatograms of the lemon EO.

Table S2. Principal compounds identified in orange EO through GC-MS analysis.

No	Compound	Retention Time (min)	Peak Area (%)
1	Ethyl butyrate	6.11	0.06
2	Hexanal	6.17	0.01
3	ϱ-Pinene	9.46	0.53
4	Sabenene	10.6	0.42
5	β-Pinene	10.81	0.04
6	β-Myrcene	10.98	1.86
7	Octanal	11.45	0.25
8	δ-3-Carene	11.7	0.16
9	D-limonene	12.71	94.44
10	Octanol	13.54	0.05
11	Linalool	14.49	0.48
12	Nonanal	14.62	0.03
13	Limonene oxide1	15.67	0.06
14	Limonene oxide2	15.8	0.06
15	Citronellal	16.12	0.03
16	4-Terpineol	17.21	0.01
17	α-Terpineol	17.65	0.1
18	Decanal	17.78	0.23
19	Neral	18.87	0.06
20	Geranial	19.75	0.03
21	Perillaldehyde	20.57	0.01
22	α-Copaene	22.5	0.01
23	Dodecanal	23.75	0.03
24	β-Caryophyllene	24.38	0.02
25	β-Copaene	24.63	0.02
26	Valencene	26.36	0.33
27	δ-Cadinene	26.93	0.03

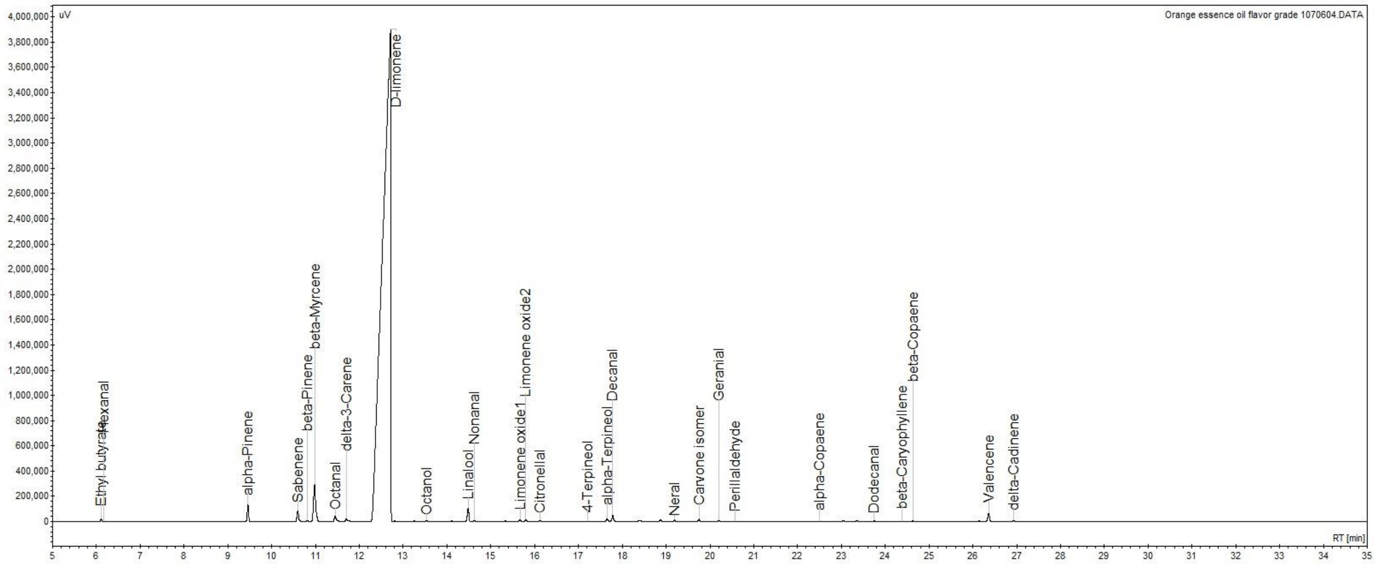

Figure S2. GC-MS chromatograms of orange EO.

Table S3. The MICs of lemon and orange EOs against various seafood-associated bacteria.

**Microorganisms**	**MIC (μL/L)**	
	**Lemon EO**	**Orange EO**
V. parahaemolyticus	2352	1176
V. harveyi	4704	2352
P. damselae	2352	588
S. putrefaciens	588	588
